# Bipolar disorder and multiple sclerosis

**DOI:** 10.1192/j.eurpsy.2021.640

**Published:** 2021-08-13

**Authors:** A. Maamri, H. Ghabi, H. Zalila

**Affiliations:** 1 Faculty Of Medincine Of Tunis, RAZI HOSPITAL, TUNIS, Tunisia; 2 Psychiatry, Outpatient Service, Razi Hospital, Manouba, Tunisia; 3 Outpatients Ward Of Psychiatry, Razi hospital, Manouba, Tunisia

**Keywords:** bipolar disorder, Multiple sclerosis

## Abstract

**Introduction:**

Multiple sclerosis (MS) is an inflammatory demyelinating illness characterized not only by severe neurological symptoms and somatic signs but also by psychiatric symptoms. Psychiatric comorbidity is common in MS. However, the incidence of psychiatric comorbidity remains understudied.

**Objectives:**

To discuss the relationship of psychiatric disorder to neurologic dysfunction in MS through a clinical case.

**Methods:**

Presentation of a clinical case of bipolar disorder in a 45-year-old woman with MS, followed by a literature review.

**Results:**

We reported a case of a 45-year-old woman, who was followed in neurology for MS since the age of twenty-five. She was stable under monthly treatment. She was referred by her neurologist for psychomotor excitement, insomnia, feeling of well being, and sexual disinhibition. The symptoms were present for three weeks. At the interview, she was euphoric, disinhibited, she had logorrhea and did not verbalize delirium. An attack of multiple sclerosis was ruled out. The patient did not report any history of psychiatric illness, epilepsy, head trauma, or use of psychoactive substances. We retained the diagnosis of bipolar disorder (manic episode). Divalproex sodium and olanzapine were prescribed with significant improvement of symptoms.

**Conclusions:**

This reported case is interesting since it highlights the possible association between multiple sclerosis and bipolar disorder. Further investigations are needed to identify potential shared risk factors between these pathologies to improve patients’ outcomes.

